# Daily Measurements from Cardiac Implantable Electronic Devices to Assess Health Status

**DOI:** 10.3390/diagnostics14232752

**Published:** 2024-12-06

**Authors:** Eva Roseboom, Fenna Daniëls, Michiel Rienstra, Alexander H. Maass

**Affiliations:** 1 Department of Cardiology, University Medical Center Groningen, University of Groningen, 9713 GZ Groningen, The Netherlands; f.daniels@umcg.nl (F.D.); m.rienstra@umcg.nl (M.R.); a.h.maass@umcg.nl (A.H.M.); 2 Department of Cardiology, Isala Hospital, 8025 AB Zwolle, The Netherlands

**Keywords:** pacemaker, implantable cardioverter-defibrillator, pacing threshold, intracardiac signal, impedance, heart failure

## Abstract

Cardiac implantable electronic devices (CIEDs) such as pacemakers and implantable cardioverter-defibrillators (ICDs) are increasingly used in the aging population. Modern CIEDs perform daily measurements, mainly aimed at discovering early signs of battery depletion or electrode dysfunction. Changes in thresholds, intracardiac signals, and pacing impedances can be caused by exacerbation of existing conditions or novel clinical problems. Pacing percentages and heart rate histograms can be used to optimize pacemaker programming, but can also be a measure of altered cardiac health status. Several measurements, such as thoracic impedance and patient activity, have been added to inform practitioners about worsening heart failure. In addition, remote monitoring of daily CIED measurements may accommodate for the prevention of the deterioration of clinical conditions. In this review, we discuss the evidence base of CIED algorithms and suggest how to use standard daily measurements to monitor the cardiac and extracardiac health status of patients with CIEDs.

## 1. Introduction

A steady increase in the implantation of cardiac implantable electronic devices (CIEDs), such as pacemakers and implantable cardioverter-defibrillators (ICDs), has occurred over recent decades, driven by large clinical trials and expanding indications in guidelines [1,2]. With the global population aging due to increasing life expectancy, a significant and exponential increase in the prevalence of patients with CIEDs is anticipated in the future. Hence, it is essential to have a fundamental understanding of the normal functioning of CIEDs, as well as the ability to identify and troubleshoot any potential CIED malfunctions or potential deterioration of clinical situation of patients with CIEDs. CIEDs integrate automated lead-monitoring alerts and other diagnostic algorithms, primarily intended to identify lead failure and evaluate battery status. Nonetheless, these device-based diagnostic tools offer physicians a plethora of additional information on cardiac function and clinical status. Additionally, these tools also provide valuable insights into cardiac function and clinical status through parameters such as pacing thresholds, intracardiac signals, lead impedance, pacing percentages, activity levels, and heart rate dispersion. The continuous evolution of CIED technology has been accompanied by the rise in remote monitoring systems, which have transformed patient care by enabling continuous, automated surveillance of device performance and patient cardiac health. Remote monitoring offers significant clinical benefits, including early detection of deterioration, reduced hospitalizations, and lower mortality rates, particularly in heart failure patients. The IN-TIME trial assessed the impact of daily implant-based telemonitoring on patients with heart failure who recently received a 2-lead ICD or 3-lead cardiac resynchronization therapy (CRT)-D. After one year, the telemonitoring group had significantly better clinical outcomes, including lower rates of worsened composite scores and fewer deaths [3]. A recent meta-analysis on home telemonitoring systems in heart failure patients was conducted, involving 36,549 patients. The meta-analysis showed a significant 16% reduction in all-cause mortality, a 19% reduction in first heart failure hospitalizations, and a 15% reduction in total heart failure hospitalizations when home telemonitoring systems were compared to standard care [4]. Remote monitoring for pacemakers is recommended to reduce the number of in-office follow-ups in patients who have difficulties attending in-office visits, and, in cases of a device component that has been recalled or is on advisory, to enable the early detection of actionable events in patients [1]. In the context of ICDs, remote monitoring is recommended to reduce the incidence of inappropriate shocks [5]. The HRS/EHRA/APHRS/LAHRS Expert Consensus Statement on Practical Management of the Remote Device Clinic states that remote monitoring for patients with CIED is standard of care [6]. Key recommendations for effective remote monitoring programs include prompt patient enrollment and adequate staffing with qualified personnel. Alert-based remote monitoring, reliant on continuous connectivity, can extend intervals between in-office device interrogations, optimizing patient care. Manufacturers have adopted various strategies to optimize remote monitoring, including daily data transmissions, event-triggered alerts, and advanced filtering algorithms. Each approach aims to address the challenges associated with the large volumes of data generated by CIEDs. However, the practical integration of remote monitoring into routine clinical workflows remains limited by resource constraints, including the need for dedicated medical personnel to manage the data effectively. Remote monitoring systems also play a critical role in reducing the frequency of in-office visits, particularly for patients with mobility issues or limited access to healthcare facilities. Additionally, they are highly effective in the early detection of device-related complications, such as malfunctions or advisories. In this context, we advocate a step-by-step approach for device interrogation to utilize all information that CIEDs provide for optimal device and patient functioning. To illustrate the step-by-step interrogation approach, several cases are presented.

## 2. Pacing Threshold

The pacing threshold (output), denoting the minimum electrical energy required to initiate cardiac contraction, is quantified in volts (V) and pulse width in milliseconds (ms), with a typical normal range for pacing threshold ranging between 0.5 and 2.0 V. Table 1 summarizes the different measurements and references values, as well as the associated pathologies that can influence them.

Most CIEDs allow for automatic daily measurements of thresholds (‘autocapture’) and will adjust the output accordingly to reduce current drain; others rely on the manual threshold measurements that are performed and programmed during outpatient visits. Ancillary indicators indicative of pacing issues, like changes in intracardiac signals or impedance, can help substantiate the underlying issue. Pacing threshold is a critical parameter for evaluating lead integrity, as a rapid increase or non-capture (failure of an electrical pacing stimulus to initiate myocardial depolarization) can suggest lead dislodgement, lead fracture, or insulation breach and lead to life-threatening situations [7,26]. Myocardial threshold, or bathmotropy, is not static, as it fluctuates in parallel with sympathetic tone and other influencing factors. The output threshold may gradually increase due to myocardial fibrosis surrounding the tip of the lead [8]. However, non-cardiac causes, such as electrolyte imbalances, can also elevate the output threshold and potentially result in loss of capture. Hyperkalemia, for instance, can disrupt the repolarization process, leading to an increased pacing threshold [9,10]. Certain drugs can affect bathmotropic potency of cardiac tissue. A case series indicated elevated myocardial thresholds post intravenous propranolol administration [11]. Antiarrhythmic drugs like verapamil, quinidine, ajmaline, mexiletine, and procainamide have shown an ability to raise pacing thresholds [12]. Conversely, adrenergic drugs like epinephrine and norepinephrine may decrease myocardial threshold [16]. Additionally, a case report noted a temporary resolution of exit block after oral glucocorticosteroids [17]. The use of steroid-eluting electrodes within pacing leads have led to reduced stimulation thresholds [27]. Another case report described gradual increased impedance and threshold in a patient treated with doxorubicin and cyclophosphamide [13]. Two case reports describe increased pacing thresholds in CIED infection, possibly due to formation of biofilm, tissue damage, or fibrosis [14,15].

**Figure 1:**
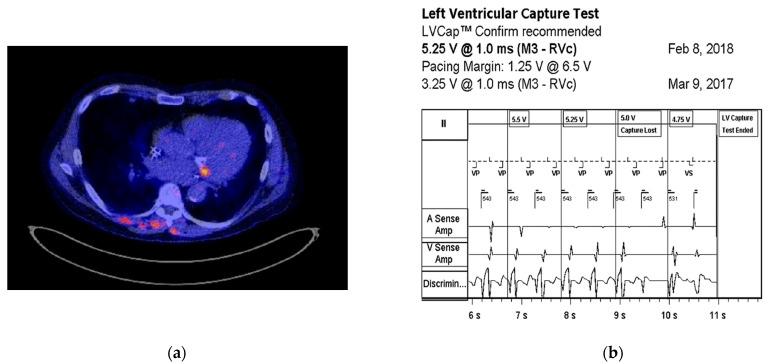
(**a**) Transverse cross-section of a PET-CT with focal FDG uptake around the left coronary sulcus, indicative of CIED infection. Additionally, there is focal FDG uptake surrounding the cervical vertebrae 4 and 5, consistent with spondylodiscitis. (**b**) Threshold test showing that the left ventricular pacing threshold increased from 3.25 to 5.25 V.

One of our cases, an adult patient with a 3-lead CRT-D, was programmed to left ventricular (LV)-only pacing mode due to an elevated threshold and non-capture of the right ventricular (RV) lead. The patient was admitted with spondylodiscitis in the context of Staphylococcus epidermidis bacteremia. Positron emission tomography-computed tomography (PET-CT) imaging revealed a localized uptake around the left coronary sulcus, indicative of LV lead infection (Figure 1a). This was further supported by device interrogation, which demonstrated an increased left ventricular (LV) lead pacing threshold of 5.25 V (Figure 1b). The patient was treated with vancomycin and rifampicin for CIED infection, and device extraction was performed. After 7 months, a new epicardial 3-lead CRT-D was implanted, and device interrogation indicated the restoration of RV and LV pacing thresholds (0.7 V and 1.4 V, respectively).

## 3. Intracardiac Signals

The sensitivity setting in a pacemaker determines the minimal amplitude of the electrical signal that the device recognizes as intrinsic activity wherein the pacing stimulus is withheld. Normal values vary on patient specifics; however, generally, a minimum value of approximately 0.5 mV is considered in the atrium and minimally 2–3 mV in the ventricle. Sensitivity can be programmed in either unipolar or bipolar mode, with bipolar pacing typically offering superior R-wave detection and reduced susceptibility to myopotential interference, even at higher sensitivity settings [28]. In contrast, unipolar pacing is more prone to external interference, particularly at lower sensitivity thresholds, making bipolar programming the preferred configuration in most cases. Sophisticated integrated algorithms analyze and interpret incoming electrical signals based on factors such as amplitude, shape, duration, and consistency. These algorithms distinguish between valid intrinsic cardiac events and noise/artifacts. Several factors influence intracardiac signals, including the lead position, which can impact its ability to detect intrinsic activity. Elevated impedance, caused by the lead’s resistance, can decrease sensitivity by attenuating electrical signals. Alterations in myocardial tissue, such as scar formation, can modify electrical properties and subsequently affect sensitivity [7]. In a case series of 35 consecutive acute myocardial infarction patients requiring temporary pacing for bradyarrhythmias, undersensing occurred in three cases associated with inferior myocardial infarction involving the right ventricle, hypothesized due to reduced intrinsic myocardial amplitude [29]. In another study that assessed the performance of ICDs in arrhythmogenic right ventricular cardiomyopathy and hypertrophic cardiomyopathy, a significant reduction in R-wave amplitude was observed within the arrhythmogenic right ventricular cardiomyopathy cohort approximately three years post-implantation [18]. The observed reduction in R-wave amplitudes suggests a potential association with the development of fibrotic tissue. Additionally, metabolic changes like hyperkaliemia can alter the amplitude of intrinsic activity, at least in canine studies, in which the infusion of potassium chloride led to a significant decrease in ventricular amplitude and slew rate, thereby impacting sensitivity [19]. Some ICD manufacturers offer the option of integrated bipolar sensing (distal coil to tip), where the inter-electrode distance can go up to ~60 mm, instead of true bipolar sensing (tip–ring), with an ~8 mm distance in between the electrodes. Integrated bipolar sensing provides the advantage of improved sensitivity due to its larger surface area; however, this can potentially lead to oversensing of R- and T-waves, myopotentials, or electromagnetic interference [30]. In a cohort of 28 patients comparing true bipolar to integrated bipolar sensing, there were no significant differences in R-wave amplitudes in sinus rhythm at baseline and 3 months after implantation, or in the detection time of induced ventricular fibrillation at implant [31].

**Figure 2:**
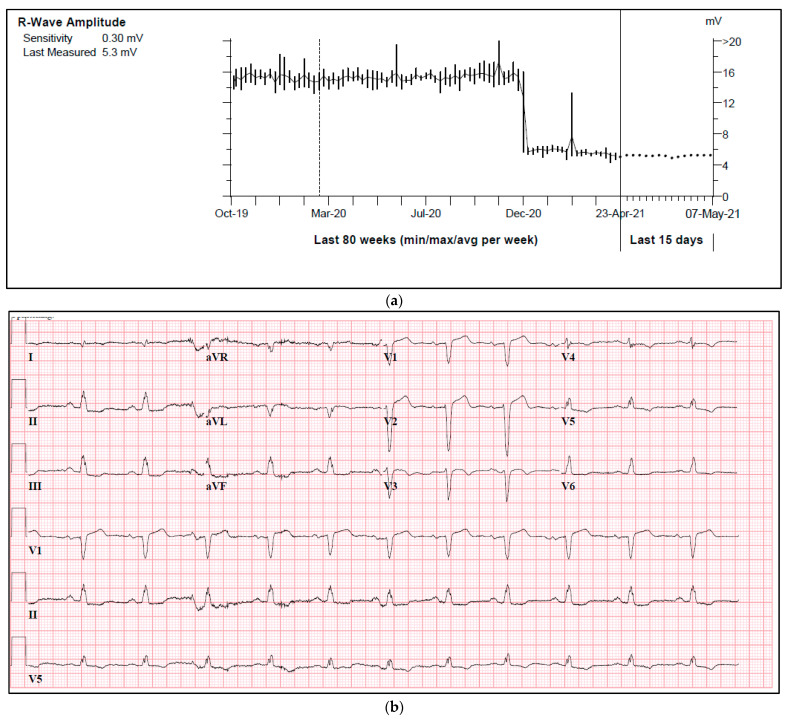

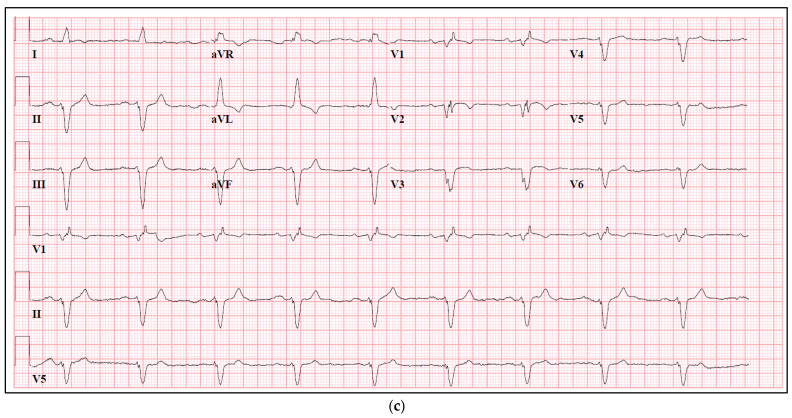
(**a**) A sudden drop in R-wave amplitude caused by new onset left anterior fascicular block. (**b**) ECG prior to a drop in R-wave amplitude. (**c**) ECG after a drop in R-wave amplitude.

One of our cases was an adult patient with ischemic heart failure and a single lead ICD. During a routine check-up, a sudden drop in R-wave amplitude was observed in a true bipolar lead, with a reduction from 15 mV to 5 mV (Figure 2a). Both threshold and impedance remained stable. Further investigation revealed the presence of a new left anterior fascicular block on the ECG, which resulted in a change in the depolarization waveform and subsequently led to a decrease in R-wave amplitude (Figure 2b,c).

## 4. Pacing Impedance

Lead impedance refers to the resistance to electrical current in the conductor that connects the electrode to the myocardium and is measured in Ohms. Pacemaker electrodes typically have an impedance ranging from 300 to 1000 Ohms, depending on the type of lead. In bipolar leads, the impedance of the ring electrode, due to its larger tissue contact area and higher resistance compared to the tip, generally presents higher impedance values. In general, the impedance of ICD shock coil spans between 25 and 100 Ohms. Amongst shock leads, single coil leads normally exhibit higher impedance values compared to dual coil leads [8]. Follow-up of impedance is crucial for assessing lead integrity. Abrupt increases in impedance may indicate a conductor fracture, which adds resistance to the circuit. Conversely, an insulation breach creates a parallel current, resulting in a decrease in lead impedance [8]. Another case report has indicated that an abrupt change in impedance can occur in conjunction with both hyperkalemia and acute renal failure, albeit the mechanisms are not yet fully understood [21]. The observed changes in pacing impedance and thresholds may be explained by several physiological pathways. Hyperkalemia, commonly associated with kidney failure, could alter the resting membrane potential of cardiac myocytes, reducing their excitability and leading to increased pacing thresholds. Additionally, chronic kidney disease-related systemic inflammation and myocardial fibrosis may contribute to increased impedance by disrupting cardiac conduction properties. An additional factor leading to increased impedance involves the development of mineralization on the tip or ring of the electrode, resulting from calcium deposition [20]. This can occur several years after the implantation of the lead, and the impedance tends to gradually increase over an extended period of time. Replacement of such leads would only be reasonable when sensing or pacing is impaired. Although impedance measurements can provide diagnostic information, it is important to note that a normal impedance trend does not exclude lead failure. A retrospective study comparing the efficacy of the lead integrity alert (LIA, Medtronic) algorithm with conventional impedance alerts revealed that in 77% of cases involving lead fracture, impedance levels remained within the normal range. To meet the criteria for LIA activation, at least two out of three criteria, including abrupt changes in pace-sense impedance, frequent extremely short R-R intervals, and rapid non-sustained ventricular tachycardia episodes, need to be fulfilled within a 60-day period. In contrast, conventional impedance alerts were activated when the impedance value reached below 200 or above 2000 Ohms [32].

**Figure 3:**
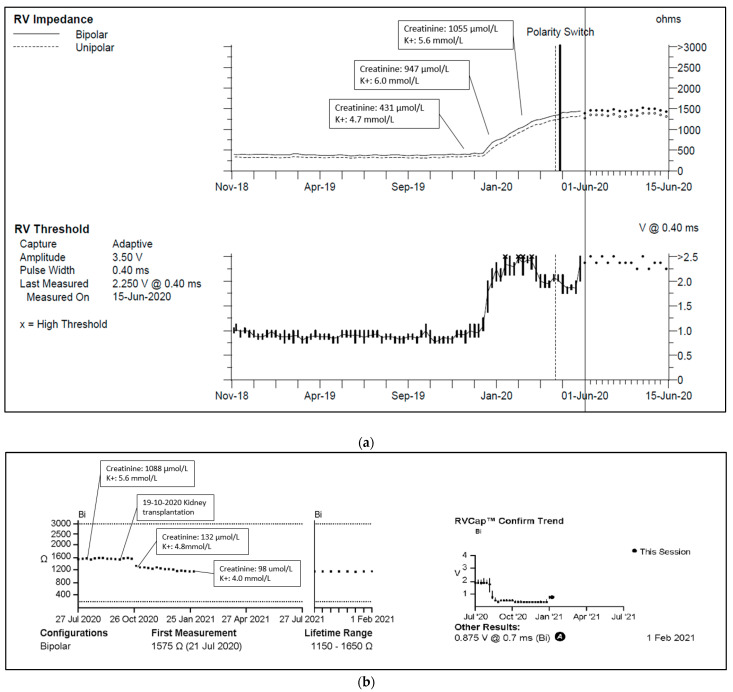
(**a**) Right ventricular lead impedance trend. Abbreviations: RV = right ventricle. Reference range: plasma creatinine 50–90 μmol/L, plasma potassium 3.5–5.0 mmol/L. (**b**) Right ventricular lead impedance and threshold 1 year trend. Reference range: plasma creatinine 50–90 μmol/L, plasma potassium 3.5–5.0 mmol/L.

One of our cases, an adult patient with a history of kidney failure and a 3-lead CRT-P. As kidney function deteriorated, plasma creatinine concentrations increased to 854–1156 μmol/L (reference value 50–90 μmol/L), and plasma potassium concentrations often exceeded 5.2 mmol/L (reference value 3.5–5.0 mmol/L). Concurrently, RV lead pacing impedance rose from 399 to 1330 Ohms, and the pacing threshold increased from 0.625 V to 2.00 V, with unchanged sensitivity (Figure 3a,b). Following a kidney transplant one year later, kidney function and plasma potassium concentrations normalized, leading to reduced pacing impedance and normalized pacing thresholds.

## 5. Thoracic Impedance

Thoracic impedance measurements detect heart failure by assessing the electrical resistance of chest tissues, which increases during congestion. Unlike the other parameters described, which are primarily associated with the device’s core functions, thoracic impedance is specifically utilized for the detection of heart failure and serves a distinct role in monitoring clinical status. Different manufacturers employ various algorithms and techniques for this measurement. OptiVol system (Medtronic) estimates fluid status through daily thoracic impedance measurements, monitoring trends over time and alerting physicians to significant changes. Boston Scientific pacemakers utilize the HeartLogic Heart Failure Diagnostic, combining heart sounds, respiration rate, activity level, and thoracic impedance to generate a heart failure index score for detecting changes. Abbott employs the Thoracic Impedance Trend Alert, monitoring thoracic impedance trends and sending alerts for significant changes that may indicate heart failure. Biotronik monitors thoracic impedance multiple times per hour and transfers the mean values daily. Several clinical studies have investigated the effectiveness of using thoracic impedance measurements for the early detection of heart failure. The FAST study, a double-blinded, prospective, multicenter study, aimed to evaluate the ability of OptiVol in Medtronic CRT-D or ICD to detect fluid accumulation in comparison to daily weight monitoring [22]. The study aimed to reduce heart failure events such as hospitalization, emergency department visits, and unscheduled clinic visits. The investigator found that intrathoracic impedance monitoring, compared to weight monitoring, had a significantly higher sensitivity (76% versus 23%, respectively) and lower false alarm rate in predicting heart failure events. It is reasonable to state that weight measurement is not a highly sensitive method to monitor fluid status or congestion, and therefore these results should be interpreted with caution [33]. The DOT-HF trial, a larger prospective randomized trial investigating the benefit of OptiVol alarm versus the control arm, in which the physician was uninformed of the thoracis impedance measurements, showed no significant difference in the composite of all-cause mortality and heart failure hospitalization [34]. The OptiVol alarms even triggered an increase in the number of outpatient visits, without correlation to signs or symptoms of heart failure. The effectiveness of the HeartLogic Heart Failure Diagnostic system was evaluated in the MultiSENSE trial. A study revealed that the algorithm demonstrated the ability to detect 70% of heart failure events on average 34 days prior to the manifestation of clinical symptoms [35]. Additionally, the study identified that the algorithm exhibited a low false-positive rate, signifying that it generated minimal false alarms. Thoracic impedance measurements lack high specificity for congestion in heart failure, as alterations in the electrical resistance of chest tissues induced by other pathologies can compromise its diagnostic accuracy. An example is pneumonia, as detailed in a case report [23].

**Figure 4:**
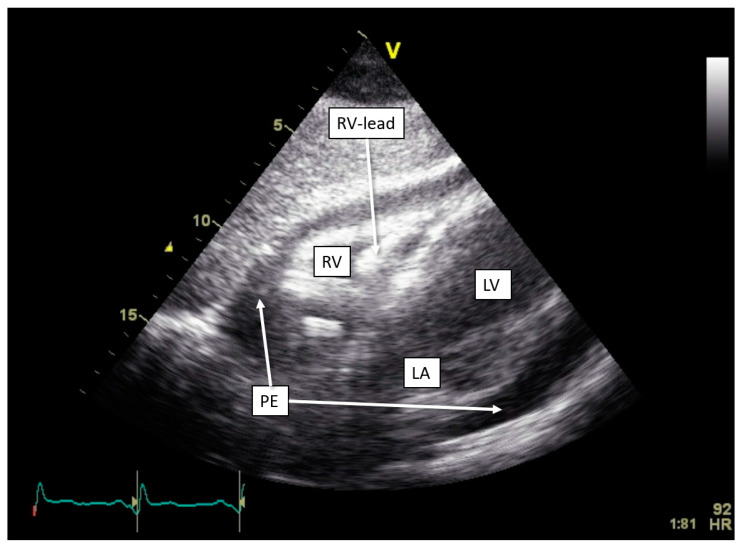
Development of pericardial effusion in OptiVol (Medtronic) alert. Transthoracic echocardiogram with subcostal view, showing pericardial effusion measuring approximately 20 mm circumferentially. Abbreviations: LA = left atrium, LV = left ventricle, PE = pericardial effusion, RV = right ventricle.

One of our cases, an adult patient with recently repositioned RV and LV lead after spontaneous dislocation of both leads, was admitted with sharp pain upon breathing. Device interrogation detected an OptiVol alarm and non-capture of the RV lead with a programmed output of 2 V. A transthoracic echocardiogram revealed pericardial effusion of 20 mm without signs of tamponade (Figure 4). Both RV lead perforation and reactive pericarditis were considered in the differential diagnosis. Treatment with colchicine and non-steroidal anti-inflammatory drugs resulted in a reduction in the pericardial effusion. Due to a persistently elevated pacing threshold, the RV lead was repositioned, resulting in the normalization of the threshold. Subsequent device interrogation has remained stable ever since.

## 6. Pacing Percentage

Pacing percentage refers to the percentage of cardiac cycles in which the device delivers pacing stimuli to either the atria or ventricles, and is a vital metric as it reflects the extent to which the patient’s intrinsic electrical conduction system is able to maintain appropriate cardiac rhythm. The ideal pacing percentage varies depending on the specific clinical context and patient characteristics. For RV pacing, this consists of aiming to minimize artificial pacing and maximize intrinsic conduction to avoid pacing-induced deterioration of left ventricular function. In the DAVID trial, patients with ischemic heart failure and left ventricular ejection fraction <40% without pacing indications were randomly assigned to receive a 2-lead ICD device programmed to DDDR-70 (dual-chamber pacing and sensing with rate response, with a lower rate of 70 bpm) or VVI-40 (ventricular pacing and sensing with ventricular inhibition, with a lower rate of 40 bpm). They found a correlation between the combined endpoint heart failure hospitalizations or death and percentage RV pacing of >40% [36]. Certain pacing algorithms, designed to minimize ventricular pacing, promote atrial pacing with ventricular backup. If AV conduction is lost, the device transitions to DDDR or DDD mode, automatically reverting back to AAI mode upon detecting the restoration of AV conduction. Furthermore, AV hysteresis, temporarily extending the AV delay after a sensed or paced ventricular event, encourages intrinsic conduction through the AV node before ventricular pacing. Sudden changes in pacing percentage should be approached cautiously as they may indicate intrinsic conduction deterioration, use of antiarrhythmics or negative dromotropic/chronotropic drugs, acute metabolic or electrolyte abnormalities like hyperkalemia or hypokalemia disrupting cardiac conduction, or intentional programming changes [24]. Regular assessments of pacing percentages allow for individualized device programming, achieving an optimal balance between pacing and intrinsic conduction. Such an approach maximizes battery life, preserves myocardial function, prevents arrhythmias, and enhances patient well-being.

**Figure 5:**
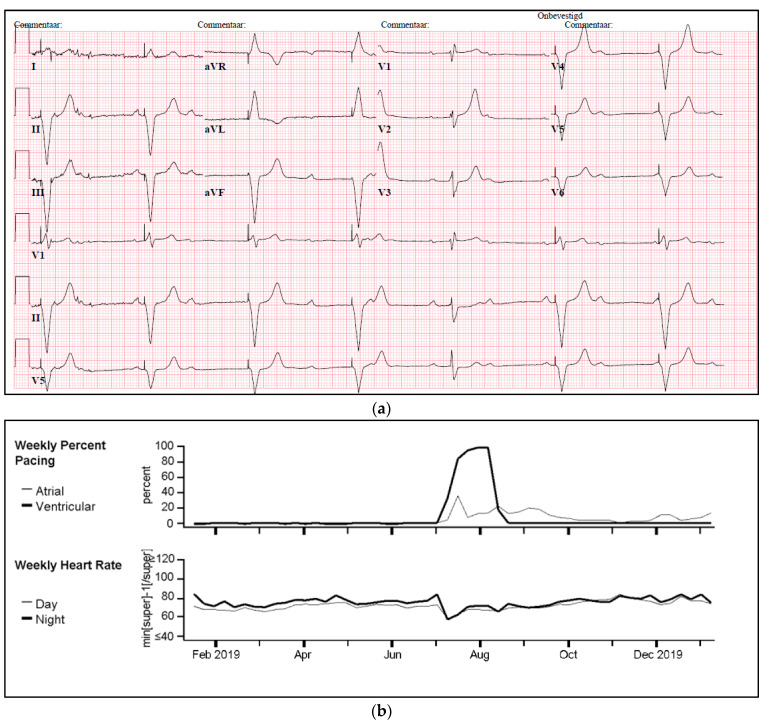

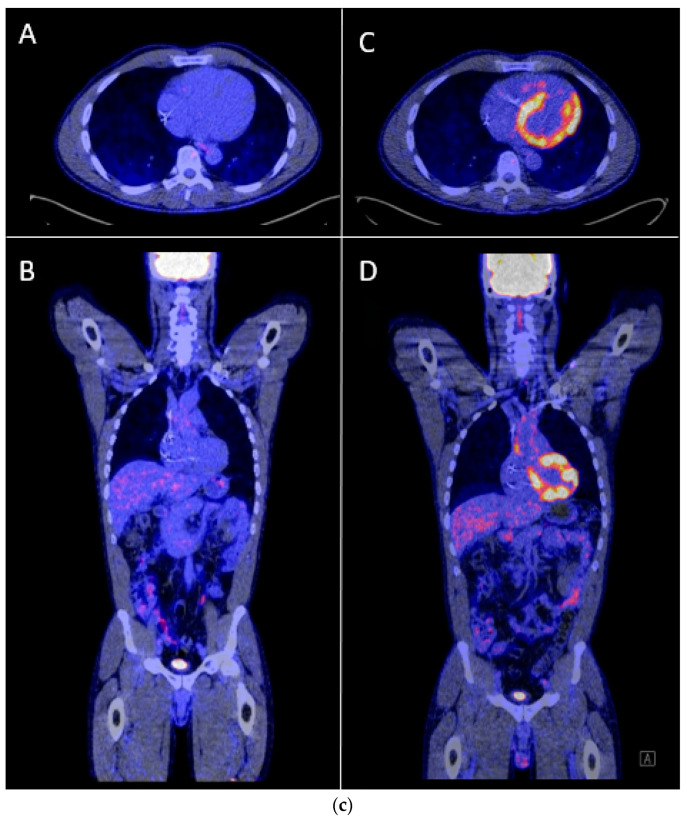
(**a**) Development of atrioventricular (AV) block and ventricular pacing at lower rate. ECG Showing sinus rhythm at 80/min, third degree atrioventricular block, ventricular pacing at 30/min. (**b**) Device interrogation showing 100% ventricular pacing. Weekly percentage pacing and weekly heart rate, 1 year trend. A sudden rise to 100% pacing is seen. (**c**) F-18 FDG PET-CT whilst in remission versus recurrent episode of cardiac sarcoidosis. (**A**,**B**): F-18 FDG PET-CT transverse and coronal cross-sections showing no signs of active cardiac sarcoidosis. (**C**,**D**): F-18 FDG PET-CT transverse and coronal cross-sections showing high suspicion of reactivation of cardiac sarcoidosis with F-18 FDG uptake of the left ventricle and interventricular septum.

One of our cases was an adult patient with third-degree AV block caused by cardiac sarcoidosis, for which he had received a 2-lead ICD programmed in DDI-40 (dual-chamber pacing and sensing with inhibition, pacing both the atrium and ventricle, with a lower rate of 40 bpm). Over a period of seven years, remission was sustained, supported by 0% atrial or ventricular pacing and the absence of rhythm abnormalities. Subsequently, the patient presented with palpitations identified as recurrent AV block, leading to 100% ventricular pacing (Figure 5a,b). PET-CT imaging revealed a high suspicion of reactivation of cardiac sarcoidosis, with increased uptake of F-18 fluorodeoxyglucose (FDG) observed in the left ventricle, septum, and right ventricular free wall (Figure 5c). Following treatment with prednisone, remission was achieved, and the percentage pacing returned to 0%.

## 7. Patient Activity

CIED-monitored activity tracking provides real-time data on a patient’s physical activity, assisting in the assessment of detecting early signs of decompensation and potential worsening heart failure, treatment effectiveness, drug adjustments, and personalized exercise recommendations, while eliminating the subjective and unreliable nature of self-reporting. Evidence suggests that exercise is beneficial for patients with heart failure, and CIEDs can aid in monitoring and tracking these activities [37,38]. A recent prospective cohort study of 94,739 individuals with accelerometer-measured light-intensity physical activity showed a significant higher risk for developing heart failure compared to those who undertook moderate- and vigorous-intensity activities [25].

**Figure 6:**
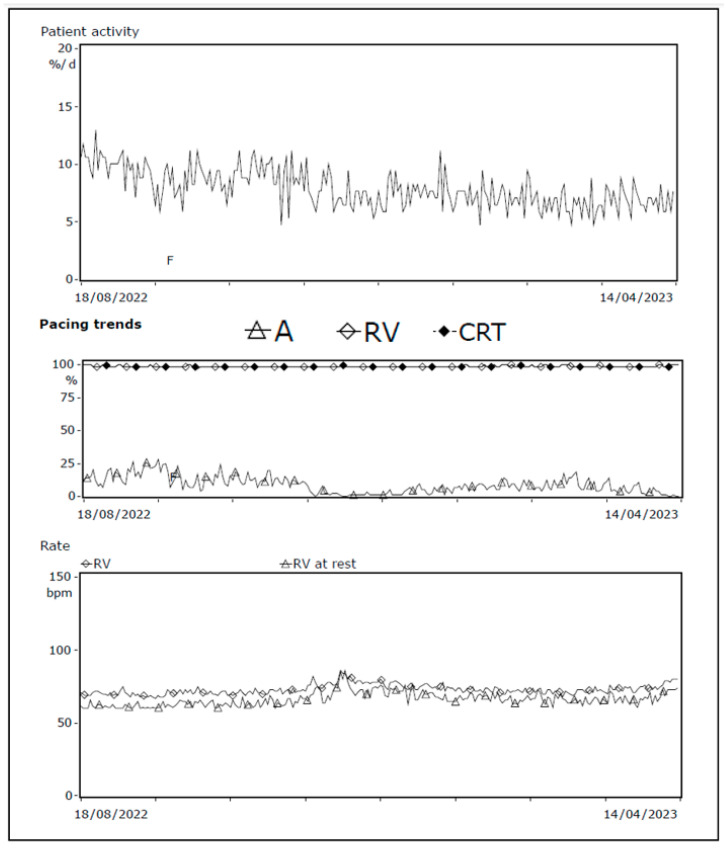
Decrease in patient activity and atrial pacing; increase in resting heart frequency. Device interrogation showing patient activity 1 year trend, pacing trends, and heart rate at rest. A decline in patient activity and atrial pacing combined with slight elevated heart rate at rest is seen, consistent with the symptoms of severe anemia. Abbreviations: A = atrial, CRT = cardiac resynchronization therapy, RV = right ventricle.

One of our cases, an adult patient with a history of ischemic heart failure and CRT-D, reported dyspnea upon exertion and fatigue during routine device checkup. Device interrogation showed no changes in impedance, threshold, or sensitivity, and biventricular pacing remained stable at 99%. However, it did reveal a decline in patient activity percentage, reduced atrial pacing (indicating higher intrinsic sinus node frequency), and a slightly elevated resting heart rate (Figure 6). During the visit, the patient appeared pale, prompting blood testing that revealed severe anemia (Hb 2.8 mmol/L, reference value 8.5–11.0 mmol/L). Further evaluation confirmed acute myeloid leukemia, and the patient underwent indicated treatment.

## 8. Arrhythmia Episodes

Arrhythmia episodes detected by CIEDs, such as tachycardias, bradycardias, or rhythm abnormalities, can indicate altered disease state necessitating intervention to reduce symptoms, risk of sudden cardiac death, or prevent ICD shocks. In addition, atrial tachyarrhythmias may increase stroke risk, and patients need to be evaluated for the initiation of anticoagulation, weighing the conflicting evidence on reducing ischemic risk and the potential for increased bleeding risk, as demonstrate in recent studies [39,40,41]. Arrhythmia episodes are discussed in detail elsewhere [5,42].

## 9. Discussion

The utility of CIEDs is expanding as the population ages and as guidelines advocate their use. This review highlights the value of CIEDs in providing extensive diagnostic information beyond basic interrogation and device diagnostics, revealing insights into the patient’s overall cardiac and extracardiac status. A systematic evaluation of data gathered through CIED interrogation highlights the breadth and clinical significance of CIED monitoring and its implications for improving patient management and outcomes.

### 9.1. Pericardial Disease

The ability of CIEDs to indirectly monitor pericardial conditions, including effusion and lead-related complications, represents a dimension in disease assessment. Our case highlights where thoracic impedance alarm provided early signs of pericardial effusion—specifically in patients with recent lead repositioning—flagging potential pericardial inflammation. However, challenges remain in interpreting thoracic impedance changes, as these readings are meant to detect pulmonary edema and lack high specificity for pericardial pathology alone.

### 9.2. Myocardial Disease

CIEDs offer a continuous method of assessing myocardial function by tracking pacing thresholds and intracardiac signals. These measurements detect myocardial changes over time, which could reflect both disease progression and therapeutic response. For example, an elevated pacing threshold often points to fibrosis or scarring within myocardial tissue, signaling a potential shift in disease status or complications due to pharmacotherapy. Studies demonstrate that tracking these thresholds allow for a more nuanced understanding of the underlying myocardial environment, as fluctuations in the threshold may be indicative of cellular-level changes that are not detectable with routine imaging. However, these measurements lack specificity. Threshold elevations might also be seen in response to ischemic events, inflammation, or even device-related complications. Thus, while CIED data are instrumental in longitudinal monitoring, its interpretation demands caution. Clinicians must consider a patient’s full clinical picture to differentiate between myocardial changes directly linked to disease versus those secondary to other factors like medications.

### 9.3. Infectious Disease

One of the primary challenges with CIEDs is managing infection risk, as these devices are associated with potential biofilm formation and lead-related infections. CIED-infections can substantially impact morbidity and mortality and often require intervention such as device extraction [43]. Device interrogation may not be the primary tool for detecting CIED-infection, but changes in pacing thresholds and lead impedance changes might serve as early indicators. By monitoring these subtle changes, clinicians may detect infections earlier than would otherwise be possible, allowing for preemptive management strategies that could avert more severe outcomes.

### 9.4. Extracardiac Disease

An area of growing interest is using CIED data to assess extracardiac health. CIEDs equipped with thoracic impedance monitoring and activity sensors can track changes that may indirectly reflect conditions such as volume overload, electrolyte imbalances, or other systemic changes. For instance, thoracic impedance can provide insight into fluid status, which is particularly useful in heart failure management. Patients with heart failure may benefit from these alerts, allowing for timely intervention before decompensation occurs. Multiple trials have attempted to demonstrate the potential of thoracic impedance monitoring to predict heart failure exacerbations and detect fluid overload early. Although promising, the evidence remains mixed, with some studies highlighting high false-positive rates that limit the specificity of these alerts. The accuracy of thoracic impedance in predicting heart failure hospitalizations was not consistently shown across studies, indicating that while the technology holds value, it may need refinement to improve clinical applicability.

## 10. Future Directions

Future perspectives in CIED management are evolving toward a more personalized, integrated approach, seeking to enhance patient outcomes and optimize healthcare workflows. One emerging frontier is personalized medicine, exemplified by cases such as the sarcoidosis patient whose pacing percentage dynamically changed with disease activity. With more advanced diagnostic algorithms, CIEDs could soon detect subtle shifts in patient-specific disease states, adapting pacing and alerting physicians proactively. This approach could be further enhanced by incorporating machine learning models that analyze each patient’s historical data to predict risks or clinical deterioration, allowing for highly individualized care plans [43,44]. Moreover, the integration of machine learning models and AI algorithms could significantly enhance the management of large volumes of data in CIED care. These technologies not only improve the identification of meaningful correlations within patient data, but also help filter out less urgent information, reducing the workload for clinicians and easing the strain on healthcare systems globally. Remote monitoring will play a critical role in this personalized care model, extending beyond routine device checks to offer continuous, real-time surveillance of device diagnostics. Current research shows that remote monitoring significantly reduces hospitalizations and improves survival rates in heart failure patients. Future systems could leverage “smart” alerting frameworks, where devices not only transmit technical data, but also include contextual alerts to attending cardiologists. These alerts would be accompanied by evidence-based recommendations for action points, like considering medication adjustments for pacing threshold shifts or assessing for infections if impedance changes arise. Such targeted notifications could be filtered by urgency and relevance, helping to prioritize patient follow-up and enabling swift interventions, thus reducing unnecessary clinic visits and optimizing in-person care time. Integrating these advancements into hospital workflows is also key. A coordinated system, where CIED alerts flow directly to electronic health records and notify the treating cardiologist, could improve the efficiency of patient management. Instead of reactive consultations based on patient-reported symptoms, clinicians would be equipped with real-time data, allowing for them to address issues preemptively. Hospitals could establish dedicated device management teams or virtual monitoring hubs to handle these alerts, ensuring continuity of care while keeping attending physicians informed. Looking further ahead, CIEDs could also incorporate wearable data, such as physical activity or sleep metrics, creating a comprehensive patient health profile. This could foster collaborative care across specialties, with insights from endocrinology, nephrology, and other fields woven into a cohesive treatment plan. By transitioning to a model that embraces personalized monitoring and proactive alert systems, CIED care could evolve to deliver more timely, precise interventions and ultimately improve quality of life for patients with complex cardiac needs.

## 11. Conclusions

Frequent assessments of CIED parameters serve a dual purpose: continuous monitoring of device functionality and insights into potential disease state changes in patients with CIEDs. We advocate for collaboration between cardiac device specialists and attending physicians, aligning device interrogation with inpatient visits for prompt responses to anomalies. Beyond apparent CIED parameters, a meticulous evaluation of additional CIED information can be important to inform personalized treatment decisions, beyond CIED functioning and arrhythmia detection and treatment.

## Figures and Tables

**Table 1:** Summary of device interrogation values and reference values.

Measurements	Reference Values	Factors Affecting Measurements and Literature References
Pacing threshold (V)	0.5–2.0 V	Increase: lead dislodgment [7], fibrosis surrounding the tip [8], hypokalemia [9,10] propranolol [11], verapamil [12], quinidine [12], ajmaline [12], mexelitine [12], doxorubicin [13], cyclophosphamide [13], and CIED infection [14,15]. Decrease: epinephrine [16], norepinephrine [16], and glucocorticoids [17].
Intracardiac signals (mV)	Atrial lead > 0.5 mV Ventricular lead > 2–3 mV	Decrease: fibrosis [7], arrhythmogenic cardiomyopathy [18], hyperkalemia [19], and left anterior fascicular block (this review).
Lead Impedance (Ohms)	200–1000 Ohms	Increase: conductor fracture [8], calcium deposition on the tip [20], hyperkalemia, and renal failure [21]. Decrease: insulation breach [8].
Thoracic impedance alert		Pulmonary edema [22], pneumonia [23], and pericardial effusion (this review).
Pacing percentage	Patient specific	Increase: conduction deterioration, negative chronotropic/dromotropic drugs, hyperkalemia [24], and hypokalemia [24].
Patient activity		Heart failure [25] and severe anemia (this review).

## Data Availability

The data presented in this study are available on request from the corresponding author due to patient confidentiality.
